# Perceptions and experiences of emergency department staff during the implementation of the four-hour rule/national emergency access target policy in Australia: a qualitative social dynamic perspective

**DOI:** 10.1186/s12913-019-3877-8

**Published:** 2019-01-30

**Authors:** Roberto Forero, Shizar Nahidi, Josephine de Costa, Daniel Fatovich, Gerry FitzGerald, Sam Toloo, Sally McCarthy, David Mountain, Nick Gibson, Mohammed Mohsin, Wing Nicola Man

**Affiliations:** 1 Simpson Centre for Health Services Research, South Western Sydney Clinical School, University of NSW, Liverpool BC, NSW 1871 Australia; 2 Ingham Institute for Applied Medical Research, Liverpool Hospital, Liverpool, NSW Australia; 3 Department of Emergency Medicine, Royal Perth Hospital, Perth, WA Australia; 4 School of Public Health and Social Work, Queensland University of Technology, Brisbane, QLD Australia; 5 Emergency Department, Prince of Wales Hospital , Randwick, NSW Australia; 6 Department of Emergency Medicine, Sir Charles Gairdner Hospital, Crawley, WA Australia; 7 School of Nursing and Midwifery, Edith Cowan University, Joondalup, WA Australia; 8 Psychiatry Research and Teaching UNit, Liverpool Hospital, NSW Health, Liverpool, NSW Australia; 9 Discipline of Emergency Medicine, University of Western Australia, Crawley, WA Australia; 10 Prince of Wales Clinical School, University of NSW, Kensington, NSW Australia; 11 School of Psychiatry, Faculty of Medicine, University of NSW, Sydney, NSW Australia

**Keywords:** Four hour rule, National Emergency Access Target, Qualitative research, Australia, Emergency department, Health policy, Unintended consequences

## Abstract

**Background:**

The Four-Hour Rule or National Emergency Access Target policy (4HR/NEAT) was implemented by Australian State and Federal Governments between 2009 and 2014 to address increased demand, overcrowding and access block (boarding) in Emergency Departments (EDs). This qualitative study aimed to assess the impact of 4HR/NEAT on ED staff attitudes and perceptions. This article is part of a series of manuscripts reporting the results of this project.

**Methods:**

The methodology has been published in this journal. As discussed in the methods paper, we interviewed 119 participants from 16 EDs across New South Wales (NSW), Queensland (QLD), Western Australia (WA) and the Australian Capital Territory (ACT), in 2015–2016. Interviews were recorded, transcribed, imported to NVivo 11 and analysed using content and thematic analysis.

**Results:**

Three key themes emerged: *Stress and morale*, *Intergroup dynamics*, and *Interaction with patients*. These provided insight into the psycho-social dimensions and organisational structure of EDs at the individual, peer-to-peer, inter-departmental, and staff-patient levels.

**Conclusion:**

Findings provide information on the social interactions associated with the introduction of the 4HR/NEAT policy and the intended and unintended consequences of its implementation across Australia. These themes allowed us to develop several hypotheses about the driving forces behind the social impact of this policy on ED staff and will allow for development of interventions that are rooted in the rich context of the staff’s experiences.

## Background

Following the implementation of the Four-Hour Target in the United Kingdom [1–3], the Western Australian (WA) Government introduced the “Four-Hour Rule (4HR)” in 2009, which was followed across Australia in 2012 by the “National Emergency Access Target (NEAT)” policy (subsequently referred to as 4HR/NEAT) [4–6]. The aim of 4HR/NEAT was to reduce Emergency Department (ED) overcrowding and access block (otherwise known as boarding) [7–10] by mandating that ED processes were completed within four hours for most ED patient presentations.

Various strategies have been tried to improve efficiency and monitor patient flow in the ED and reduce access block [11–15]. At its peak, in 2008, access block affected between 40 and 70% of admitted patients, and prolonged length of stay in ED before reaching in-patient beds [16]. However, the principal cause of access block, namely impeded access to inpatient beds, is generally not controlled by ED staff. Thus, the introduction of 4HR/NEAT was partly intended to ensure a whole of hospital approach (WoHA) or whole of system approach to address the issue [17].

The introduction of the Four-Hour target has generated several intended and unintended consequences, with different and oppositional reactions amongst stakeholders during their implementation [18–22]. While recent studies have investigated the impact of 4HR/NEAT on patient outcomes, the socio-psychological impacts on ED staff have not been previously explored [10, 23, 24].

This article is part of a series of manuscripts reporting the results of this project. The aim of this study was to describe the psychosocial impact of the policy by identifying the intended and unintended consequences of the Difussion of Innovation Theory in relation to the impact of 4HR/NEAT [25]. The theory categorises the consequences in three different ways: anticipated or unanticipated, desirable or undesirable, and direct or indirect [18, 19]. This study is relevant for policy makers and emergency medicine clinicians who may be considering large-scale policy changes in the local environment.

## Methods

The methodology of this qualitative study has been recently circulated in *BMC Health Services Research* [26]. We have also published results on system changes and outcomes [27, 28]. As discussed in the methodology paper, this qualitative study is part of a large data linkage study exploring the overall impact of the introduction of the 4HR/NEAT. The main objective of the current study was to explore the impact of the implementation of the 4HR/NEAT on ED staff at the individual, peer-to-peer, inter-departmental and staff-patient levels.

As indicated in the methods paper [26], we used several sampling techniques to recruit 119 ED staff, from 16 participating hospitals across New South Wales (NSW), Queensland (QLD), Western Australia (WA) and Australian Capital Territory (ACT). Study participants were mostly health professionals. We applied an integrated interview protocol (containing demographic information and pre-identified open-ended questions) to conduct semi-structured interviews. The interviews were audio recorded, transcribed and imported into NVivo V.11 [29]. We used a combination of thematic and content analyses to identify the issues of concern to ED staff.

### Data analysis

The analysis was carried out in three stages. The first stage comprised theme identification and the development of a conceptual framework (see acknowledgements). The second stage comprised elements of content and thematic analysis to compare participants’ experience by role and location. We chose this approach because of the exploratory nature of the project investigating how ED staff defined, used and adapted to the policy changes. These were coded and compiled in a codebook (see acknowledgments). After the conceptual framework was developed/designed [26], we proceeded to explore several theory-driven approaches to describe the impact of the policy including the Diffusion of Innovation Theory[19].

**Fig. 1:**
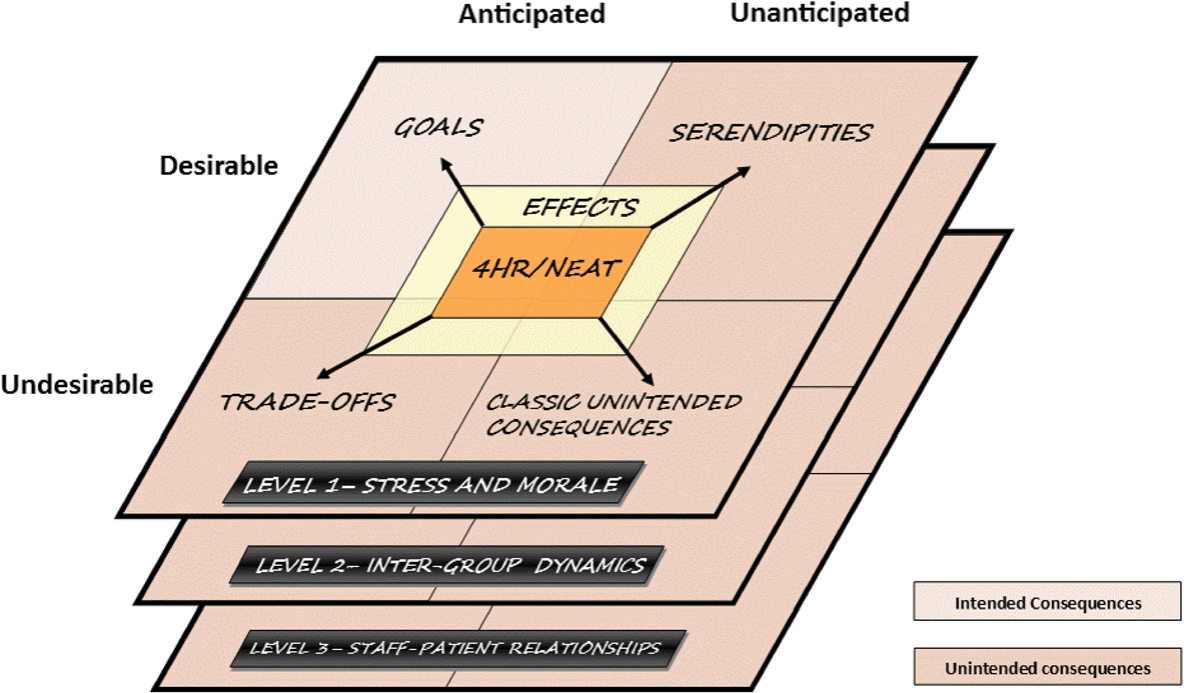
Classification of the consequences according to Diffusion of Innovations model [25] adapted for the Impact of the 4HR/NEAT policy at the individual, interpersonal and interaction with patients’ level

The third stage explored the associated effects and consequences described above. We found that the diffusion of innovation theoretical approach was well suited for drawing out the factors to be considered when explaining the impact of a large-scale policy. An important element of the suitability of the theory was that we reached theoretical saturation for most categories [26]. We then adapted the diffusion of innovation framework and recoded the main concepts in NVivo to explore how the different levels of ED operation were affected, namely the personal, intergroup dynamics and staff patient relationships (levels 1–3, Fig. 1) [25]. For each theme, results were presented in the number of interviewees, the percentage of interviewees indicating the specific themes, total number of quotations for each item and mean number of quotations per interviewee. The proportion test was used to compare the significant differences in percentages across staff roles and states.

#### Ethics approval

We received ethical approval from the respective Human Research Ethics Committees of WA Department of Health (DBL.201403.07), Cancer Institute NSW (HREC/14/CIPHS/30), ACT Department of Health (ETH.3.14.054) and QLD Health (HREC/14/QGC/30) as well as site specific approval from participating hospitals.

## Results

A total of 119 ED staff members were interviewed, of whom 62 (52%) were women, 44 (37%) nurses, 43 (36%) physicians, 21 (18%) directors (also physicians), and 11 (9%) administrative staff. There were 52 (44%) participants from NSW/ACT, 37 (31%) from QLD and 30 (25%) from WA.

We identified three levels of social impact in accordance with the Diffusion of Innovation Theory [25] corresponding to the main themes as illustrated in Fig. 1 and Table 1.

**Table 1:** Themes and categories based on the Diffusion of Innovations model [25] reported by participants

Themes	Categories of 4HR/NEAT Consequences			
	Anticipated and desirable (GOALS)	Unanticipated and desirable (SERENDIPITIES)	Anticipated and Undesirable (TRADE-OFFS)	Unanticipated and Undesirable (UNINTENDED CONSEQUENCES)
Personal experiences of stress and morale	• 4HR/NEAT improved the clinical role performance (8; 11)	• 4HR/NEAT improved morale in ED staff (18; 52) • 4HR/NEAT decreased stress (4; 4)	• 4HR/NEAT increased workload (81; 419)	• 4HR/NEAT increased stress and decreased morale (109; 1147)
Intergroup dynamics	• 4HR/NEAT improved relationships with rest of the hospital (33; 40) • 4HR/NEAT signified the importance of hospital’s executive buy-in (21; 59) • 4HR/NEAT necessitated the Whole of Hospital Approach (WoHA) (87; 334)	• 4HR/NEAT improved communications within ED staff (29; 50) • 4HR/NEAT improved ED teams and teamwork (25; 39) • 4HR/NEAT increased autonomy of ED staff (16; 25)		• 4HR/NEAT undermined ED teams and teamwork (35; 82) • 4HR/NEAT worsened communication within ED staff (26; 43) • 4HR/NEAT shifted the power in decision making from ED to hospital executives (6; 7) • 4HR/NEAT impaired relationships with rest of the hospital (77; 257) • Hospital failed to employ the WoHA (54; 190) • Suboptimal leadership and insufficient buy-in at hospital executive confounded 4HR/NEAT-related changes (47; 128)
Interaction with patients	• 4HR/NEAT improved staff-patient communication (26; 56)		• 4HR/NEAT had no change on staff-patient relationships (17; 20)	• 4HR/NEAT decreased staff-patient communication (43; 140) • Non-4HR/NEAT factors influencing staff-patient communication (6; 6)

NOTE: The numbers in brackets represent interviews and quotations per theme; (# interviews; # quotations)

### Theme one – Personal experiences of stress and morale

**Fig. 2:**
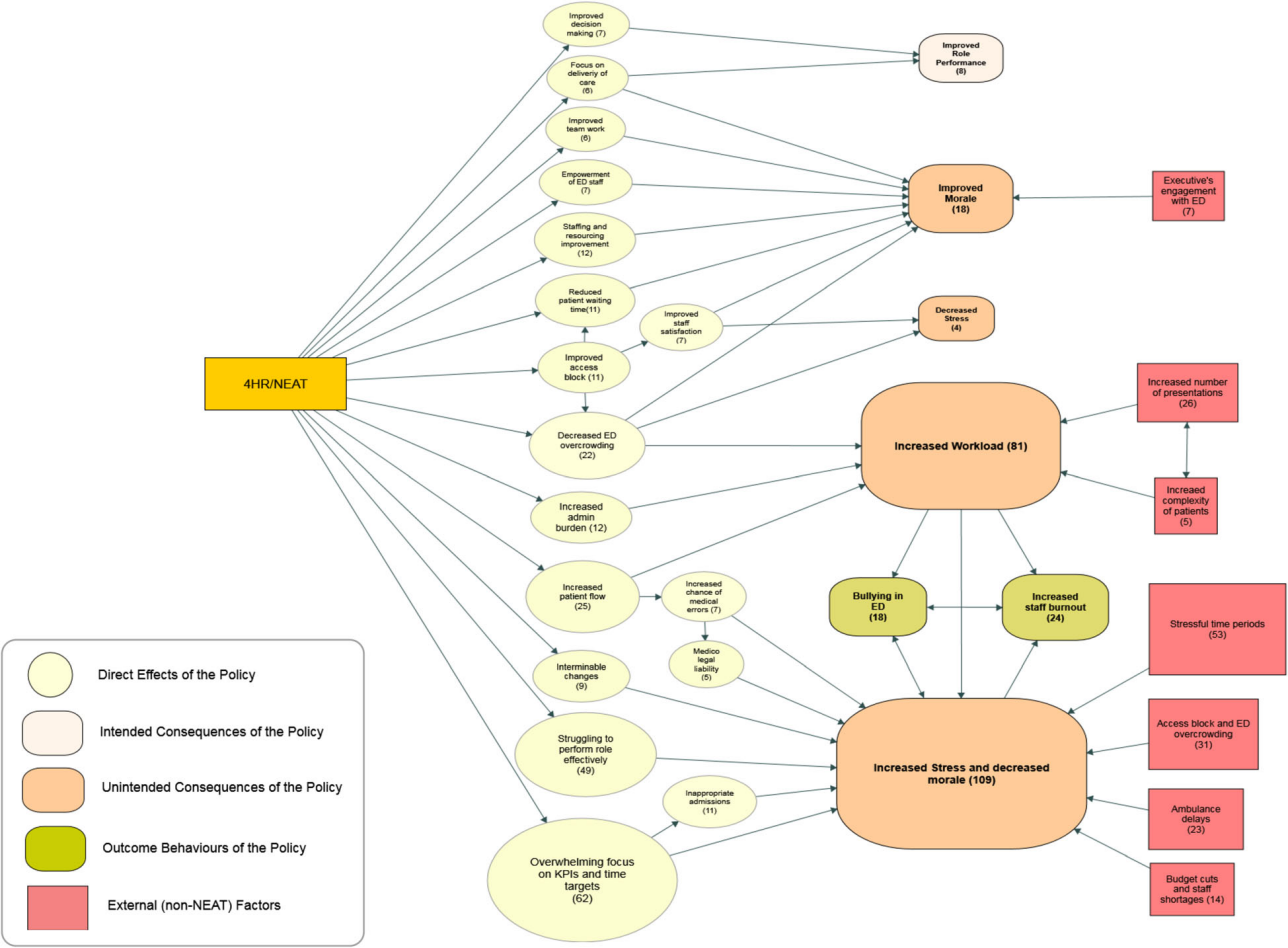
Direct effects and intended/ unintended consequences of the 4HR/NEAT policy in relation to stress and morale. The numbers in brackets represent the number of participants/interviews

The 4HR/NEAT policy generated a spectrum of negative and positive emotions ranging from high stress, inability to cope and low morale, to enthusiasm and satisfaction with professional performance. Figure 2 illustrates the diagram of change of the direct effects and the Diffusion of Innovations consequences at this level.

Most of the participants stated that one of the anticipated and desirable impact of the 4HR/NEAT (Goals) was the improvement of clinical role performance. This was attributed to a more efficient decision-making process and enhanced ability to focus on patient care. Some participants also reported unanticipated and desirable impact on stress and morale (Serendipities) leading to improved morale in ED (because 4HR/NEAT reduced overcrowding, waiting time, and increased staff satisfaction - Fig. 2). Other participants indicated that the 4HR/NEAT decreased stress through reduction of access block and overcrowding:

*“It made a far less congested ED environment… there was less apathy associated with trying to do things in a reasonable timeframe. Previously, there was such a barrier to movement of patients that it was quite a heart sink, and it would be day after day after day, and invariably we’d have, you know, poor elderly patients that were stuck on trolleys for prolonged periods, and it was increasingly, over that decade, a demoralising experience to be in ED, and it became our world” (ED Director, WA).*

Some participants also reported that the policy improved communication between ED staff, ED team work and autonomy. In relation to anticipated and undesirable outcomes (Trade-Offs), 81 (61%) participants reported that their workload increased in association with the 4HR/NEAT and a minority reported that the policy did not change staff-patient relationships. In relation to the unanticipated and undesirable outcomes (unintended consequences), participants reported a substantial change associated with increased stress and decreased morale. A large number of participants also reported that 70% of nursing staff and 74% of doctors were affected by workload-driven stress. Four participants indicated that the 4HR/NEAT policy increased administrative burden, therefore generated more responsibility without additional diagnostic support for speedier decisions.

As indicated in Tables 2 and 3, the comparison and contrast analysis revealed that 109(92%) participants reported increased stress and decreased morale (unintended consequence, Table 1). This was higher in WA compared to NSW/ACT and QLD. Paradoxically, 28 (23%) participants suggested that improvements in staffing and resourcing from 4HR/NEAT policy contributed to morale improvement (serendipity, Table 1). These resources were perceived to have alleviated excessive ED workloads, especially ED physical layout changes or new equipment. They also reported that empowering ED staff to control the admission process improved decision making, focus on delivery of care and team work, enhancing morale. The 4HR/NEAT also created further engagement with patients and increased autonomy of ED staff (Fig. 2).

**Table 2:** Comparison of the emergent key concepts and number of quotations across different states

Theme	Key Concepts	Number and % of respondent for each concept by States							Number and mean number of quotations by States						
		All states (*n*=119)	NSW/ACT (*n*=52)		WA (*n*=30)		QLD (*n*=37)		All states	NSW/ACT		WA		QLD	
		No.	No.	%	No.	%	No.	%	No.	No.	Mean	No.	Mean	No.	Mean
Personal experiences of stress and morale	4HR/NEAT increased stress and decreased morale	109	44	85%	30	100%	35	95%	1146	323	7.3	512	17.1	311	8.9
	4HR/NEAT increased workload	81	28	54%	22	73%	31	84%	419	135	4.8	132	6.0	152	4.9
	4HR/NEAT improved morale in ED staff	18	7	13%	7	23%	4	11%	52	13	1.9	32	4.6	7	1.8
	4HR/NEAT improved the clinical role performance	8	0	-	7	23%	1	3%	11	0	-	9	1.3	2	2.0
	4HR/NEAT decreased stress	4	1	2%	2	7%	1	3%	5	1	1.0	2	1.0	2	2.0
Intergroup dynamics	4HR/NEAT necessitated the Whole of Hospital Approach (WoHA)	87	31	60%	28	93%	28	76%	334	120	3.9	124	4.4	90	3.2
	4HR/NEAT impaired relationships with rest of the hospital	77	33	63%	21	70%	23	62%	257	86	2.6	84	4.0	87	3.8
	Hospital failed to employ the WoHA	54	26	50%	9	30%	19	51%	190	113	4.3	13	1.4	64	3.4
	Suboptimal leadership and insufficient buy-in at hospital executive confounded 4HR/NEAT-related changes	47	21	40%	9	30%	17	46%	128	59	2.8	18	2.0	51	3.0
	4HR/NEAT undermined ED teams and teamwork	35	13	25%	14	47%	8	22%	82	36	2.8	31	2.2	15	1.9
	4HR/NEAT improved relationships with rest of the hospital	33	15	29%	10	33%	8	22%	40	15	1.0	14	1.4	11	1.4
	4HR/NEAT improved communications within ED staff	29	11	21%	16	53%	2	5%	50	18	1.6	29	1.8	3	1.5
	4HR/NEAT worsened communication within ED staff	26	9	17%	13	43%	4	11%	43	16	1.8	22	1.7	5	1.3
	4HR/NEAT improved ED teams and teamwork	25	5	10%	12	40%	8	22%	39	9	1.8	17	1.4	13	1.6
	4HR/NEAT signified the importance of hospital’s executive buy-in	21	11	21%	6	20%	4	11%	59	32	2.9	16	2.7	11	2.8
	4HR/NEAT increased autonomy of ED staff	16	9	17%	4	13%	3	8%	25	16	1.8	4	1.0	5	1.7
	4HR/NEAT shifted the flow of power from ED to hospital executives	6	6	12%	0	-	0	-	7	7	1.2	0	-	0	-
	4HR/NEAT led to overwhelming pressure from department of health	4	0	-	2	7%	2	5%	5	0	-	3	1.5	2	1.0
Interaction with patients	4HR/NEAT decreased staff-patient communication	43	12	23%	14	47%	17	46%	140	36	3.0	46	3.3	58	3.4
	4HR/NEAT improved staff-patient communication	26	9	17%	6	20%	11	30%	56	15	1.7	23	3.8	18	1.6
	4HR/NEAT had no change on staff-patient relationships	17	6	12%	7	23%	4	11%	20	8	1.3	8	1.1	4	1.0
	Non-4HR/NEAT factors influencing staff-patient communication	6	2	4%	3	10%	1	3%	6	2	1.0	3	1.0	1	1.0

**Table 3:** Comparison of the emergent key concepts and number of quotations across different ED staff roles

Theme	Key concepts	Number and % of respondent for each concept by ED staff roles									Number and mean number of quotations by ED staff roles								
		All (*n*=119)	Physician (*n*=43)		Admin (*n*=11)		Nursing (*n*=44)		Director (*n*=21)		All	Physician		Admin		Nursing		Director	
		No	No	%	No	%	No	%	No	%	No	No	Mean	No	Mean	No	Mean	No	Mean
Personal experiences of stress and morale	4HR/NEAT increased stress and decreased morale	109	39	91%	10	91%	42	95%	18	86%	1146	458	11.7	42	4.2	519	12.4	127	7.1
	4HR/NEAT increased workload	81	32	74%	6	55%	31	70%	12	57%	419	180	5.6	16	2.7	179	5.8	44	3.7
	4HR/NEAT improved morale in ED staff	18	10	23%	0	0%	3	7%	5	24%	52	27	2.7	0	0.0	6	2.0	19	3.8
	4HR/NEAT improved the clinical role performance	8	4	9%	1	9%	3	7%	0		11	7	1.8	1	1.0	3	1.0	0	0.0
	4HR/NEAT decreased stress	4	4	9%	0		0		0		5	5	1.3	0	0.0	0	0.0	0	0.0
Intergroup dynamics	4HR/NEAT necessitated the Whole of Hospital Approach (WoHA)	87	34	79%	4	36%	31	70%	18	86%	334	133	3.9	21	5.3	135	4.4	45	2.5
	4HR/NEAT impaired relationships with rest of the hospital	77	30	70%	4	36%	27	61%	16	76%	257	108	3.6	10	2.5	90	3.3	49	3.1
	Hospital failed to employ the WoHA	54	19	44%	4	36%	17	39%	14	67%	190	65	3.4	5	1.3	59	3.5	61	4.4
	Suboptimal leadership and insufficient buy-in at hospital executive confounded 4HR/NEAT-related changes	47	17	40%	1	9%	20	45%	9	43%	128	66	3.9	1	1.0	34	1.7	27	3.0
	4HR/NEAT undermined ED teams and teamwork	35	13	30%	2	18%	17	39%	3	14%	82	28	2.2	3	1.5	45	2.6	6	2.0
	4HR/NEAT improved relationships with rest of the hospital	33	14	33%	2	18%	13	30%	4	19%	40	18	1.3	2	1.0	16	1.2	4	1.0
	4HR/NEAT improved communications within ED staff	29	11	26%	5	45%	11	25%	2	10%	50	16	1.5	10	2.0	19	1.7	5	2.5
	4HR/NEAT worsened communication within ED staff	26	9	21%	2	18%	12	27%	3	14%	43	12	1.3	3	1.5	22	1.8	6	2.0
	4HR/NEAT improved ED teams and teamwork	25	8	19%	1	9%	13	30%	3	14%	39	14	1.8	1	1.0	20	1.5	4	1.3
	4HR/NEAT signified the importance of hospital’s executive buy-in	21	10	23%	3	27%	7	16%	1	5%	59	22	2.2	6	2.0	25	3.6	6	6.0
	4HR/NEAT increased autonomy of ED staff	16	8	19%	1	9%	5	11%	2	10%	25	16	2.0	1	1.0	5	1.0	3	1.5
	4HR/NEAT shifted the flow of power from ED to hospital executives	6	4	9%	0		1	2%	1	5%	7	4	1.0	0	0.0	1	0.0	2	2.0
	4HR/NEAT led to overwhelming pressure from department of health	4	2	5%	0		1	2%	1	5%	5	3	1.5	0	0.0	1	1.0	1	1.0
Interaction with patients	4HR/NEAT decreased staff-patient communication	43	21	49%	2	18%	17	39%	3	14%	140	67	3.2	6	3.0	57	3.4	10	3.3
	4HR/NEAT improved staff-patient communication	26	14	33%	1	9%	8	18%	3	14%	56	36	2.6	2	2.0	13	1.6	5	1.7
	4HR/NEAT had no change on staff-patient relationships	17	6	14%	2	18%	5	11%	4	19%	20	7	1.2	2	1.0	6	1.2	5	1.3
	Non-4HR/NEAT factors influencing staff-patient communication	6	1	2%	1	9%	3	7%	1	5%	6	1	1.0	1	1.0	3	1.0	1	1.0

Another factor (goal) mentioned by 41 participants (40%), in Tables 1 and 2, indicated that appropriate executive engagement improved morale (e.g., presence of hospital executives on the floor and management-level consultation). When comparing across states and roles, it appeared that references to morale improvement was higher in WA than NSW and QLD.

Four participants reported that EDs relied on junior and inexperienced staff, who were more likely to experience burnout, which would increase the stress on senior staff who would be affected subsequently (Table 3).

An ED nurse from a WA hospital explained:

*“Stress levels were incredibly high … all the RMOs had to talk to the registrars in a certain amount of time, in a timeframe, they’d formulate their notes which, as a junior doctor, was very, very difficult under pressure because they’re junior, and getting a story articulated on paper and then giving it to their registrar was a really hard thing for them to do within a short period of time.” (ED Nurse, WA).*

One in five participants (25, 21%), reported that a constant flow of patients through ED contributed to increased workload, pushing them to continuously work at a very fast pace (Fig. 2). A large number of QLD and NSW/ACT participants reported that increased presentations and patient complexity exacerbated the problem and contributed to increased difficulty complying with the target.

Sixty-two (52%) participants indicated that the 4HR/NEAT generated an overwhelming focus on Key Performance Indicators (KPIs), time targets and constant changes that made them struggle with their role (Fig. 2). In several NSW/ACT and QLD hospitals, the 4HR/NEAT policy was viewed by executives and inpatient teams as exclusively an ED target. In some WA hospitals, a ‘please explain’ attitude towards poor performance was perceived as a punitive measure on ED. Some participants from NSW/ACT and WA also indicated that medical staff were pressured by nursing staff to achieve time targets. It was also mentioned that pressure to admit may have increased the chance of inappropriate and unnecessary hospital admissions.

Many ED nurses and physicians across all locations, especially in WA, described a prevailing stress-generating perception among ward staff who tended to believe that ED staff could not perform all their duties effectively, because they *“were not able to order multiple diagnostic tests, neither could consider all differential diagnoses.” (ED Nurse, QLD).*

Increased stress in some QLD and WA hospitals was reported to be associated with lack of clarity about 4HR/NEAT processes and role expectations. Some participants indicated that the 4HR/NEAT policy required a multitude of changes in the ED, making it hard for staff to work:

*“… We did the Four-Hour Rule, a renovation, and swine flu. So, it’s very hard to separate them because they were all interrelated, and yeah, so it is hard to compartmentalise them. So, during the NEAT it was really challenging … It almost felt like a bit of a “get out of gaol free” card in some perspectives, because the stuff – we had to change. We had to get on with it and we had to adjust, and that, and that adaptation had to be quick, and it was changing all the time. So, it got chaotic and confusing.” (ED Nurse, WA).*

Five participants mentioned that faster ED processing, rapid assessment and decision-making can increase the chance of medical errors and legal liability concerns. Other factors such as long or inconvenient working hours, staff shortages, budget cuts and increased sick leave were suggested to contribute to increased levels of stress. Many participants also indicated that ambulance delays were important in generating stress in the ED and hindered satisfactory clinical performance that potentially undermined delivery of care:

*“… The ambulance service… they introduced a 20-minute offload policy here, so we have to get a patient off their stretcher within 20 minutes. Either onto a stretcher, into our waiting room, or into a bed, or they just leave the patient. So that was an added stress to the Emergency Department. So, other hospitals are able to ramp. It’s a term that they talk about … So that’s been an impact. And you know, patients didn’t particularly like being put in the corridor, and there’s no privacy.” (ED Nurse, NSW/ACT).*

Lastly, there were reports of increased bullying in the ED after the 4HR/NEAT introduced in NSW/ACT and WA. Nurses and junior ED doctors were more likely to be bullied. Fourteen (11%) participants reported that they were either bullied themselves or observed inappropriate behaviours, such as treating others in an overbearing, harassing or intimidating manner about 4HR/NEAT policy compliance:

*“There’s quite a lot of threat and bullying that goes on around [the 4HR/NEAT policy] compliance, particularly towards nursing staff. I mean, they are immensely threatened by the staff above them to perform and are forced to do things that I’m sure they know within themselves are the wrong thing to do.” (ED Physician, NSW/ACT).*

### Theme two – Intergroup dynamics

**Fig. 3:**
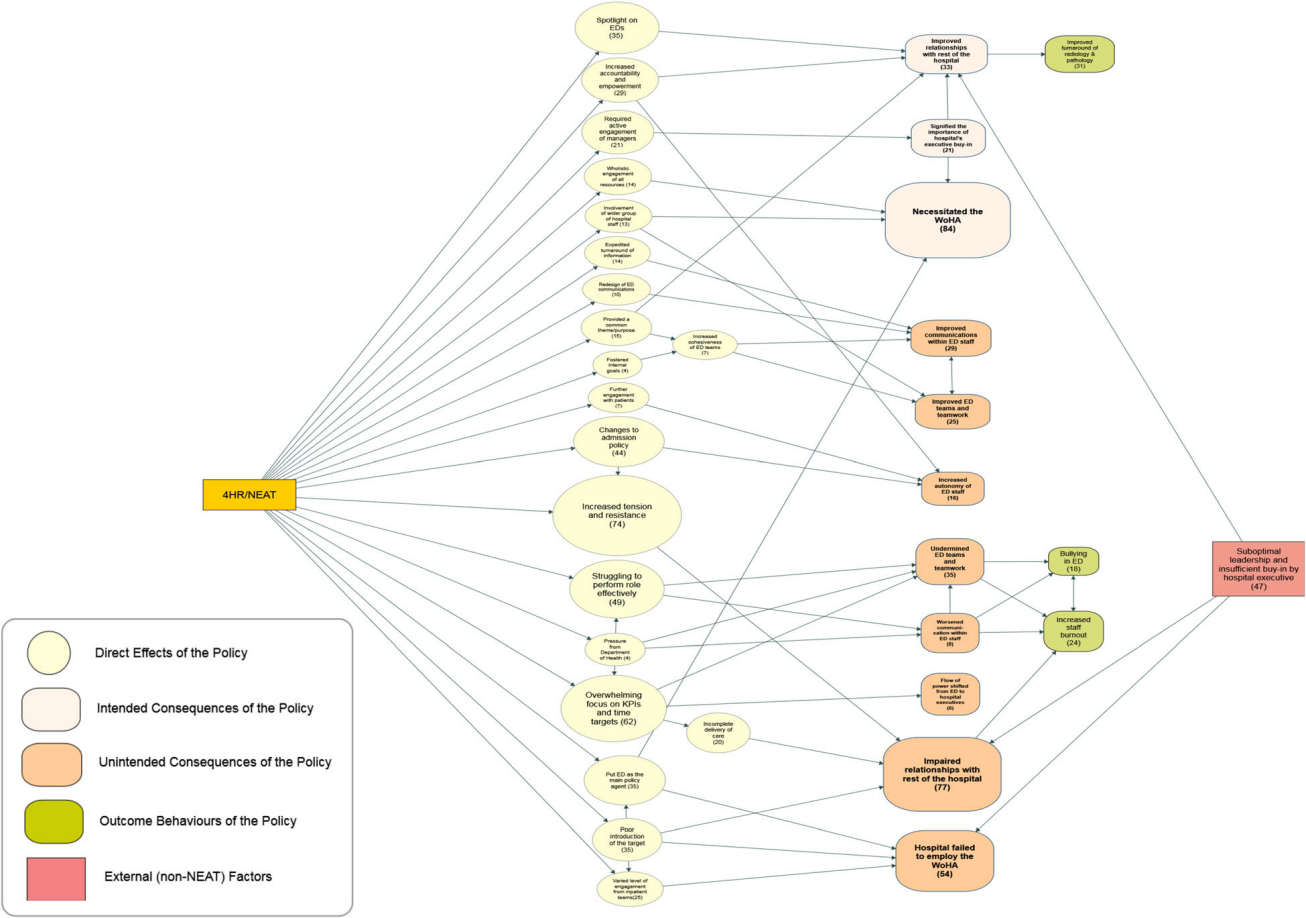
Direct effects and intended/unintended consequences of the 4HR/NEAT policy in relation to intergroup dynamics. The numbers in brackets represent the number of participants/interviews

In terms of intergroup dynamics, the 4HR/NEAT influenced the dynamics within ED, the dynamics between ED and other departments and the interactions between ED and hospital executives. Three prevailing concepts that emerged from our analysis:

In relation to the Goals, a majority of the participants reported that the policy improved relationships with the rest of the hospital and had resulted in significant hospital executives buy-in, with an improved whole of hospital approach. Firstly, participants indicated that the 4HR/NEAT policy improved communication between ED staff (e.g., doctors, nurses, department head) which they perceived was positively influenced by the redesign of ED communications and improved turnaround of information time. The most dominant influence on ED teamwork was increasing the ‘cohesiveness of ED teams’ (Fig. 3).

Keeping the focus of everyone on the target brought ED staff together as one team and created opportunities for involvement of a wider group of staff to foster efficient and pro-active communication which improved relationships with the rest of the hospital (Fig. 3).

*“It’s given us a bit more power in the hospital... It’s enabled us to make disposition decisions and, yeah, empowered us as a department to say, “This patient is admitted.” Previously you might say, “Oh, I think they should be admitted under cardiology,” and then the cardiology registrar would rock down and go, “they should be under respiratory,” and then you would go into this “argy-bargy” of finding an inpatient team.” (ED Physician, NSW/ACT).*

Despite these, there were statements by physicians suggesting worsening of ED cohesion and poorer communications between nurses and doctors:

*“I think it degrades relationships within the department. It makes the nurses constantly question the doctors about what they’re doing – which is not necessarily a bad thing, but again, it just makes a very conflicting level of interaction rather than a collegial level of interaction.” (ED Physician, NSW/ACT).*

According to Fig. 3, the 4HR/NEAT policy also generated increased tension and resistance in the ED environment that was manifested by ‘tense’, ‘fractious’ and ‘discouraging’ interpersonal communications, which sometimes led to staff burnout and bullying. In Fig. 3, we also noted that 22 participants suggested that ED staff burnout was manifested by high rates of short and long-term absenteeism and resignations during the 4HR/NEAT implementation. While few participants explicitly mentioned bullying, there were discussions about deteriorating staff relationships (especially between junior ED staff and senior inter-departmental ward staff). Other interviewees suggested that the 4HR/NEAT created an overwhelming focus on key performance indicators (KPIs), undermining their teamwork (Fig. 3). Some nurses also reported feeling additional pressure and the perceived tendency to focus on compliance more than doctors. Several participants indicated that the 4HR/NEAT created tension between nursing managers and experienced nurses unhappy with the level of care that they could provide.

In addition to these, the pressure from state health departments to meet targets made some EDs struggle to perform effectively, and consequently undermined their teams and teamwork (Fig. 3):

*“The Department of Health and the government, as a mistake, in my opinion, called it Emergency Access Targets, which still makes everyone think it’s an Emergency Department problem. It should be called Hospital Access Targets. And almost all emergency nurses that I speak to, agree with that. If anybody else in the system is feeling pressure to get your admissions in or get discharges out, or create capacity in the hospital, they blame it on this thing that is called NEAT, but because it’s got “Emergency” in the name, they just think that we’re creating the problem, whereas if it had been called a Hospital Access Target, that would have really helped with the education for everybody.” (ED Nurse, NSW/ACT).*

Secondly, some participants indicated that increased tension between ED and other departments, impaired relationships with the rest of the hospital. These and other negative perceptions in Fig. 3 were linked to factors such as increased tension and resistance, overwhelming focus on time targets, pressure from government departments and limited inpatient team capacity to maintain patient flow.

In Fig. 3, 33 participants indicated that the 4HR/NEAT may have led to improvement in professional relationships and interactions with inpatient and other hospital teams, because it provided a common theme/purpose generating positive changes with the rest of the hospital (e.g., pathology and radiology departments that improved their turnaround time). The 4HR/NEAT policy also fostered redistribution of power differentials between ED and other wards and increased accountability and focus that made ED staff feel empowered to engage in decision-making, all of which led to the improvement in the relationships between ED and the rest of the hospital.

Also, in Fig. 3, 44 participants indicated that the 4HR/NEAT created changes in the ED admission policy. This was described as the controversial ‘one-call admission policy’ which allowed some ED’s to admit patients to inpatient wards directly. Some EDs were perceived as overbearing and/or overly pressuring some inpatient units. Seventy-four participants believed that ‘one call admission policy’ generated some tension and resistance, and 14 indicated that this policy empowered them to make decisions and to take clinical ownership of their patients.

Thirdly, participants identified that the 4HR/NEAT generated active engagement from managers that delivered buy-in (Table 1 and Fig. 3). Effective implementation and sustainability required constructive and supportive executive engagement. Satisfactory relationships between ED and hospital executives fostered hospital-wide changes requiring engagement and support of other services. While most participants believed there was no change in the flow and structure of power within ED, a few NSW/ACT participants indicated that focus on KPIs and time targets shifted power to hospital management (Fig. 3).

*“Certainly, the power has changed a lot in the last few years… once upon a time the Medical Director of the ED had a lot more power over the budget and how things – how resources were spent, and hiring and how things were done, whereas now, I think a lot of that has been taken away from the medical staff and given to hospital management, who are often nursing staff.” (ED Director, NSW/ACT).*

It was indicated that the success of the 4HR/NEAT policy was reliant on executive support for strategies which may fast track patient transfer to inpatient units such as ‘one-call’ or enabling wards to go over census [30, 31]. Some participants noted that ownership was taken by inpatient teams when hospital executives showed a spirit of communication, problem-solving and cooperation to achieve 4HR/NEAT policy targets (Table 1 and Fig. 3). They also indicated that some WoHA strategies were effective, such as holistic engagement of all resources, clinical redesign, escalation of responses to overcrowding, consultant-led inpatient units, implementing activity-based funding models and reduced turnaround times in diagnostic services:

*“My understanding from before and after, as well, is that the ED very much was its own little hub and wasn’t necessarily as cohesive to the rest of the hospital, which now has changed. It is no longer just an emergency department; it’s a whole of hospital ... Like, I work with the service manager and I send to her NEAT data monthly, so she’s very much part of the performance, how they’re going, so NEAT’s a whole of hospital – they’re being held responsible for their part as well.” (Admin staff, NSW/ACT).*

On the contrary, many staff believed that their hospital did not take a WoHA, but rather kept the focus on ED as the main 4HR/NEAT policy agent. Some indicated that their executives focused on data without fully understanding the clinical and logistic aspects of 4HR/NEAT compliance. This generated increasing expectations and psychosocial pressure on ED staff believing that “ED was responsible for any failures in implementing the 4HR/NEAT policy”. Participants also indicated that ED was unable to fulfil 4HR/NEAT requirements as meeting the target and maintaining patient flow were mainly outside ED control. For example, barriers such as “the executive not reinforcing the 4HR/NEAT policy”; “lack of inpatient-team responsibility”; and “obligations and awareness of ED limitations” required synchronised and sustainable culture change at all levels:

*“All the changes have occurred in ED, but the original purpose of NEAT was to change the practices of the whole of the hospital, not just the ED, and I think a lot of the effort that has happened in the rest of the hospital has been tokenistic rather than really engaged. Tokenistic in terms of, you know, they’re cherry-picking easy initiatives to give the impression that they’re changing their practice.” (ED Director, NSW/ACT).*

### Theme three – Interaction with patients

**Fig. 4:**
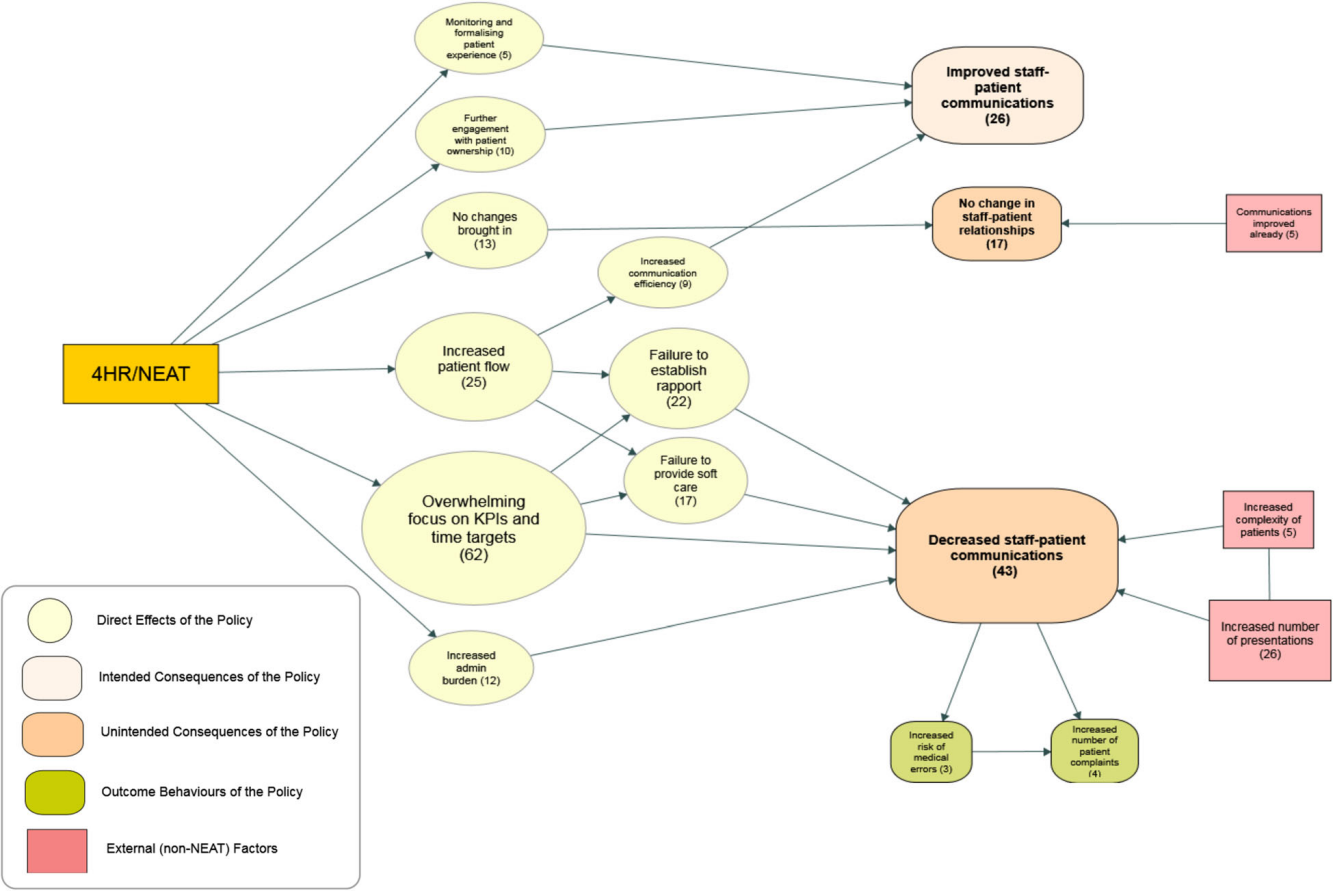
Direct effects and intended/unintended consequences of the 4HR/NEAT policy in relation to staff-patient relationships. The numbers in brackets represent the number of participants/interviews

In relation to the Goals, a few participants reported an anticipated and desirable consequence of the 4HR/NEAT being the close monitoring of patient experience and further engagement with patient ownership which improved staff-patient communication. In addition, increased patient flow also increased communication efficiency (Fig. 4). There was however, a Trade-off in relation to no changes reported by 17 participants who were influenced negatively by the 4HR/NEAT in relation to staff-patient relationships (see Table 1 and Fig. 4).

*“So, I think from a patient perspective, developing a relationship with an ED specialist who introduces themselves to you and, importantly, says that they’re responsible for your care, and I think that’s a patient requirement we have failed to meet in the past, I think that would be good.” (ED Physician, QLD).*

An unintended consequence was that some participants believed that the 4HR/NEAT had no influence on communications and interactions between staff and patients:

*“I don’t think it’s actually changed too much. I mean, we’re obviously going to spend less time with them, but I don’t think the quality has changed too much. We still do what we can, we still try to explain to them.” (ED Physician, WA).*

Another trade-off was that staff-patient communication was thought by some to have been reduced because the 4HR/NEAT implementation increased the pace of patient flow. This limited the ability of staff to establish rapport with patients. Seventeen (14%) participants reported that it reduced the capability of ED staff to provide “soft care” that may not be necessary for diagnosis but could improve the patient experience.

*“They [patients] are more treated like numbers, and not a person. It’s sort of dehumanised the patient coming through… So, from triage, you’re trying to assess whether they’re going to be admitted or not, and then really, that person is either an admission or a discharge.” (ED Nurse, WA).*

Many ED staff indicated that decreased staff-patient communication could increase the numbers of complaints in ED and may increase the risk of medical errors. Some participants indicated that the dual task of accommodating complex patients (multiple comorbidities) or high acuity patients, the increased number of patients and overcrowding compromised the relationship with patients*.*

## Discussion

In this study, we draw information from the experience and insight of ED staff during the implementation of the 4HR/NEAT policy across Australia. It was a unique opportunity to assess the impact at the personal level during the implementation period. Using the diffusion of innovations model [25], our study highlighted for the first time key intended and unintended consequences of the 4HR/NEAT policy in the ED context.

Previous studies on the dynamics of interactions between ED and other hospital departments highlighted the challenges of providing an integrated system of patient care [32, 33]. This is due to factors such as compartmentalisation of care, lack of coordination among different sections within the healthcare system, and a linear approach to the continuity of care concept [34, 35].

The implementation of the 4HR/NEAT was an initiative trying to bring together the whole system to work in an integrated and non-fragmented manner to provide patients with fast, efficient and safe care upon their presentation to the ED. A detailed description of our findings in relation to the impact of the policy on the whole of hospital approach (WoHA) has been published elsewhere [27, 28].

In general, the 4HR/NEAT policy generated both positive and negative effects on those who were at the forefront of implementing it, the ED staff. As indicated in the thematic analysis, we found strong relationships between the percentages of the participants per finding, especially in relation to the personal experience with stress and morale. The 4HR/NEAT was never intended to directly change the social milieu, but our findings clearly show the 4HR/NEAT policy had significant effects in the social domain. The 4HR/NEAT generated changes at the personal level in relation to stress and morale, which have implications in the context of health psychology (i.e., addressing bullying, stress and burnout in the ED), social policy, such as social inequities and individual stress in the ED, and social epidemiology, such as changing health staff’s behaviour in the working ED environment.

While some participants reported that the 4HR/NEAT policy reduced overcrowding and waiting time and increased staff satisfaction (Table 1 and Fig. 2); it is important to acknowledge that this consequence was not a clearly stated aim of the 4HR/NEAT, but one identified in this study.

The 4HR/NEAT policy also generated consequences for group level interactions which were relevant to organisational psychology as well as social science and epidemiology (Fig. 3). The prominence of bullying in Australian hospitals has been confirmed and recently highlighted [36]. In relation to patient consequences, they also have implications for health services research and at the health care level (Fig. 4).

Discussions about unintended consequences resulting from health policy, federal regulations and hospital policies were frequently reported by participants at different stages of the interviews. These were also relevant in the context of social policy and social epidemiology because substantial differences in stress and morale were observed across states and roles. Some of them were quoted more (see mean number of quotations, in Tables 2 and 3) in WA compared to NSW/ACT suggesting higher levels of effects in WA than NSW/ACT and QLD (Table 2). The mean number of quotations also suggested substantial differences in WA compared to NSW/ACT and QLD in relation to WoHA and staff-patient communication. In addition, Nurses and senior doctors were more likely to report increased stress and morale than ED directors and administrative staff (Table 3).

The principal weakness of the 4HR/NEAT policy was its ‘unidimensional nature’. It is a process indicator and in general process improvements often need to be weighed against their impact on cost or quality. This study has identified that the achievements against the 4HR/NEAT policy target have been associated with social costs in terms of it impacts on the social environment of the ED and on the relationships between ED staff and others (e.g. patients and other staff).

Considering that the 4HR/NEAT policy was introduced after the Four-Hour target policy in the UK, we reviewed some of the qualitative research conducted after the implementation of the policy in the UK, and we have found similar findings [37–40]. However, those studies did not have the same level of qualitative rigor as our study; and a major comparison with the UK was beyond the scope of our study.

### Strengths and limitations

As indicated in the methods [26], this is a rigorous qualitative study that allowed comparability across hospitals and ED roles without compromising the dependability of the results with high levels of saturation and inter-rater reliability. Secondly, there was a relatively large number of participants (at the national level) when compared with UK qualitative studies. Thirdly, our study achieved a high level of credibility (validity) and dependability (reliability) of the analysis which are a true reflection of the perspectives reported by the group of participants across different Australian jurisdictions. Lastly, we also achieved theoretical saturation and comparable coverage of the issues identified in relation to theory of innovation.

However, there are several limitations to our study that are worth mentioning. Firstly, the study was done after implementation of the 4HR/NEAT and so the findings were post-hoc and we could not identify events occurring before, during or after the implementation. Secondly, the main limitation is that the perceptions about the 4HR/NEAT policy are restricted to ED staff without perspectives from executives or inpatient teams also involved in policy implementation.

## Conclusion

This study has identified significant aspects around social interactions for individuals, groups and the health system that were unintended consequences of the introduction of the 4HR/NEAT policy. This included some significant negative effects on perceived stress and morale for ED staff, respectively. It was also evident that the 4HR/NEAT policy generated changes in inter-group dynamics that may have significant effects on future health care; for example change in managerial power distribution and the influence or lack of influence of clinician groups within hospitals, and inter-group dynamics, for example the positive engagement or disengagement of staff in their day to day workplace, which has been shown to influence the quality of patient care [41]. There were also potentially concerning changes in perceived interactions with patients. These findings provide a new dimension in understanding the social dynamic processes during policy change implementation in the ED environment.

## Abbreviations

4HR/NEAT: Four Hour Rule/ National Emergency Access Target
ACT: Australian Capital Territory
ED/EDs: Emergency Department(s)
HREC: Health Research Ethics Committee
KPI: Key Performance Indicator
NSW: New South Wales
QLD: Queensland
WA: Western Australia
WoHA: Whole of Hospital Approach
